# The accuracy of the Montenegro skin test for leishmaniasis in PCR-negative patients

**DOI:** 10.1590/0037-8682-0433-2019

**Published:** 2020-04-27

**Authors:** Ana Bárbara Sapienza Pinheiro, Patricia Shu Kurizky, Marina de Freitas Ferreira, Marco Antonio de Souza Mota, Jaqueline Santos Ribeiro, Edson Zuza de Oliveira, Carlos Augusto Souza, Daniel Holanda Barroso, Raimunda Nonata Ribeiro Sampaio, Ciro Martins Gomes

**Affiliations:** 1 Universidade de Brasília, Núcleo de Medicina Tropical, Brasília, DF, Brasil.; 2 Universidade de Brasília, Faculdade de Medicina, Pós-graduação em Ciências Médicas, Brasília, DF, Brasil.; 3 Universidade de Brasília, Faculdade de Medicina, Brasília, DF, Brasil.; 4 Universidade de Brasília, Faculdade de Medicina, Laboratório de Dermatomicologia, Brasília, DF, Brasil.; 5 Universidade de Brasília, Faculdade de Ciências da Saúde, Pós-graduação em Ciências da Saúde, Brasília, DF, Brasil.

**Keywords:** Leishmaniasis, Cutaneous, Mucocutaneous, Diagnosis

## Abstract

**INTRODUCTION:**

As highly specific molecular biology-based techniques may not be sensitive enough for the diagnosis of American tegumentary leishmaniasis (ATL), clinicians frequently rely on immunological tests before treatment initiation. Hence, the correct combination of diagnostic tests is imperative for ATL diagnosis. We aimed to evaluate the accuracy of the Montenegro (Leishmanin) skin test (MST) in polymerase chain reaction (PCR)-negative patients to accurately detect ATL.

**METHODS:**

Patients with a clinical picture compatible with ATL were divided into ATL (confirmed by lesion smear, culture indirect immunofluorescence, and/or histopathology) and no-ATL (diseases that can mimic leishmaniasis) groups. Conventional PCR for the minicircle kDNA of *Leishmania* was performed, and the MST was carried out for PCR-negative patients.

**RESULTS:**

Ninety-nine patients were included in this study, including 79 diagnosed with ATL (6 with mucocutaneous leishmaniasis) and 20 without ATL (no-ATL group). The MST showed a high sensitivity of 90.0% (95% confidence interval [CI] = 69.90-97.21) in PCR-negative patients that was 10% higher than the sensitivity reported in PCR-positive population (79.66%; 95% CI = 67.73-87.96).

**CONCLUSIONS:**

One of the most important reasons for PCR negativity among patients with active ATL is the presence of a strong cellular immunological response, especially in chronic and mucocutaneous leishmaniasis. This reinforces the considerable utility of the tests that detect cellular responses against *Leishmania* antigens such as the MST in PCR-negative patients when the performance in screening situations is questionable.

## INTRODUCTION

The correct diagnosis of American tegumentary leishmaniasis (ATL) is imperative, as treatment is frequently associated with important adverse reactions^1^. Considering the lack of any gold standard for the diagnosis of ATL^2^, physicians must coordinate between epidemiological, clinical, and laboratorial criteria, a strategy that is largely imperfect^2^. The Brazilian Ministry of Health has recently recognized the importance of therapeutic tests in suspected ATL cases that pose difficulty in diagnosis^3^.

Parasitological tests such as lesion smears and cultures are useful tools for ATL diagnosis but are often limited by low sensitivity^2^. Histopathological examination, in addition to parasite identification, may show expected immunological signs of ATL, such as plasma cell infiltrates and granuloma formation^2^. Immunological exams such as serology techniques and the Montenegro (Leishmanin) skin test (MST) are some of the most widely used techniques for the diagnosis of ATL^4^ ^,^ ^5^. MST is based on an intradermal reaction after the application of *Leishmania* antigens^2^, and has been long used for the initial screening and diagnosis of ATL. However, in Brazil, the availability of MST has drastically reduced owing to new sanitary regulations. In endemic areas, a positive MST result can also be attributed to previous leishmaniasis or an asymptomatic exposure to the parasite (infection)^2^.

Huge investments have been recently made in molecular biology techniques, and polymerase chain reaction (PCR) is gaining popularity. Strategies such as forensic DNA extraction, use of primer pairs to amplify the kDNA minicircle of *Leishmania* spp., and variations such as real-time-based PCR have improved the diagnosis sensitivity; however, under practical conditions, these strategies exhibit a maximum sensitivity of 90%^6^ ^-^ ^8^. Thus, a considerable number of patients may still be treated without the direct detection of parasites during diagnosis.

ATL, in its localized form, is known to induce intense cellular responses and granuloma formation. This immunological reaction may lower the parasite load in the lesion and consequently reduce the sensitivity of parasitological tests and PCR but may be insufficient to cure the disease^9^. This is particularly a problem in South America where *Leishmania (Viannia) braziliensis* infection is endemic^10^. This species is associated with the mucocutaneous form of leishmaniasis and sometimes with long-lasting clinical presentations, which are also related to low parasite loads^11^. As MST is associated with a cellular immunological response, we believe that its use in PCR-negative patients may be a rational and cost-effective strategy for the diagnosis of ATL.

In the present study, we evaluated the accuracy of MST in PCR-negative patients and compared its association with other immunological response assessments (such as the presence of granuloma in histopathological examination) to reduce the need for therapeutic tests in ATL management.

## METHODS

We performed a cross-sectional accuracy study. After protocol creation, patients with a clinical picture compatible with ATL who were evaluated at the Dermatology Division of the University Hospital of Brasília (HUB) from January 2012 to December 2015 and underwent a PCR test for skin fragments were consecutively included.

### Composite reference standard (the presently defined gold standard for ATL case definition)

ATL case was defined by positive parasite visualization in skin smears, cultures, or histopathological examination. In the absence of the abovementioned criteria, ATL positivity was defined in the form of a highly compatible inflammatory infiltrate in histopathological examination, positive indirect immunofluorescence with no evidence of other diseases using special stains or PCR for mycobacteria, and complete cure after pentavalent antimonial therapy. Although the histopathology of ATL can take various forms, the presence of granuloma and/or plasma cell infiltrates in samples without amastigote forms was considered as highly compatible with ATL.

The no-ATL group comprised patients without ATL but with diseases that have a clinical picture similar to that of ATL. In these patients, differential diagnosis was confirmed by the same exams used for case evaluation (e.g., vascular ulcers, cutaneous tuberculosis, subcutaneous mycosis, pyoderma gangrenosum, squamous cell carcinoma).

### Index tests (presently evaluated diagnostic tests)

### Polymerase chain reaction

PCR was performed for the amplification of a 120-bp sequence in the minicircle kDNA of *Leishmania spp*. using the primers 5´-(G/C)(G/C)(C/G)CC(A/C)CTAT(A/T)TTACACCCAACCCC-3´ and 5´-GGGGAGGGGCGTTCTGCGAA-3´ (Eurofins MWG Operon®, Huntsville, AL, USA)^12^. The reactions were performed on a Mastercycler® Pro thermocycler (Eppendorf®, Hamburg, Germany) at a final volume of 25 μL comprising 1x PCR buffer, 0.2 mM of each dNTP, 1.5 mM of magnesium chloride (MgCl_2_), 0.5 μM of each primer, 2.0 units of Taq DNA polymerase (Invitrogen, Foster City, USA), and 5 μL of DNA template. The amplification cycles included an initial denaturation step of 3 min and 30 s at 94°C, followed by 35 cycles at 93°C (30 s), 60°C (1 min), and 72°C (1 min), and a final extension at 72°C (10 min) and incubation at 4°C. All reactions included a negative and positive control with *L. braziliensis* culture lysates. In brief, 2 μL of the amplified product was mixed with 2 μL of a xylene-cyanol preparation (Vetec®, Duque de Caxias, Rio de Janeiro, Brazil) and 1 μL (1:100) of GelRed™ (Biotium®, Hayward, CA, USA) and the mixture was loaded onto a 2% agarose gel immersed in 1× Tris base, acetic acid, and ethylenediaminetetraacetic acid (EDTA) buffer. A 100-bp marker was used (Invitrogen®, São Paulo, Brazil). Electrophoresis was performed in a Sub-Cell® GT Cell 170-4403 horizontal tank (BIO-RAD®, Hercules, CA, USA) for 90 min at 90 V and 400 mA. The gel was visualized on an EC3 Imaging System (UVP®, Upland, CA, USA).

The subgenus was identified by PCR restriction fragment length polymorphism (RFLP) using the enzymes *Hae*III and *Bsr*1 (New England Biolabs®, Inc., Ipswich, MA, USA) after an overnight incubation at 37°C and 65°C, respectively. Fragments were then visualized using polyacrylamide gel electrophoresis^12^. All negative samples were processed using endogen C18X primers for DNA extraction, as described elsewhere^12^.

### Montenegro (Leishmanin) skin test

MST was performed using an antigen provided by the Centre for Production and Research of Immunobiológicos - CPPI, Piraquara, Paraná, Brazil. A total of 0.1 mL of the solution was intradermally injected on the anterior surface of the left forearm. The solution comprised fragments of *L. amazonensis* (WHO reference strain MHOM/BR/73/PH8). The cocktail included 40 μg/mL of protein nitrogen, 0.005 g/mL phenol, 0.0098 g/mL sodium chloride, and distilled water to make up the volume to 1 mL. The test was considered positive upon the formation of a papule of a diameter equal to or greater than 5 mm after 48 h.

### Statistical analysis

Categorical variables were compared using the chi-square test or its exact version. Numerical variables were compared using the Mann-Whitney U test. Test results were compared using the McNemar’s test or Cohen’s kappa coefficient. The percentage of positive results in patients with ATL was used to calculate sensitivity, and that of negative results in patients without ATL was used to calculate specificity. Accuracy was calculated as the sum of the true-positive and true-negative results divided by the total number of patients tested. Missing values were ignored in unpaired tests. The programmes SPSS 20.0 (IBM Corporation, Armonk, NY, USA) and SAS 9.4 (SAS Institute, Inc., Cary, NC, USA) were used for statistical analysis. Statistical significance was defined at p < 0.05, and the confidence interval (CI) was set at 95%.

### Ethics

All patients participating in the study were required to sign a written consent form. This project was approved by the Ethics Committee of the Faculty of Medicine - University of Brasília (UnB) under the protocol number 37190914.0.0000.5558.

## RESULTS

Ninety-nine patients were included in this study, of which 79 were diagnosed with ATL (6 with mucocutaneous leishmaniasis) and 20 were allocated to the control group. The demographic characteristics, including sex, age, lesion site, lesion size, and disease time, were similar between the groups (Table 1). PCR-RFLP technique detected *L. viannia* infection in 55 patients and *L. amazonensis* infection in 4 patients. The no-ATL group comprised 8 patients with subcutaneous mycosis, 6 with vascular ulcers, 3 with pyoderma gangrenosum, 2 with squamous cell carcinomas, and 1 patient with cutaneous tuberculosis.

**TABLE 1:** Demographic and basic disease characteristics of patients with American tegumentary leishmaniasis and controls.

	ATL group	No-ATL group	
	(79 patients)	(20 patients)	p-value
	n (%)	n (%)	
Gender			0.688
Male	49 (62.0)	14 (70.0)	
Female	30 (38.0)	6 (30.0)	
Lesion site:			0.066
Head	15 (19.0)	5 (25.0)	
Trunk	6 (7.6)	1 (5.0)	
Superior limbs	12 (15.2)	8 (40.0)	
Inferior limbs	38 (48.1)	6 (30.0)	
Multiple	8 (10.1)	0 (0.0)	
Age, mean (STD)	49.8 (15.27)	46.7 (16.84)	0.452
Diameter (cm), mean (STD)	3.2 (1.85)	3.3 (1.59)	0.881
Disease duration (months), median (IQR)	8.78 (16.6)	10.88 (27.8)	0.763

**ATL:** American tegumentary leishmaniasis, **STD:** Standard deviation; **IQR:** Interquartile range.

The comparison between the results of all performed exams (index tests and the composite reference standard) revealed the best accuracy for PCR, which showed 100% specificity (Table 2 and Table 3). The results of different tests, in general, showed slight agreement (Table 2). As expected, 22 patients with negative cultures, 41 negative for amastigote in histopathology, and 22 patients with negative smears were detected positive for ATL in PCR. On the other hand, only 2 patients with positive culture results, 4 positive for amastigote in histopathology, and 4 patients with positive smears were deemed ATL negative in PCR.

**TABLE 2:** Information on the diagnostic accuracy of the tests used as a composite reference standard.

	Sensitivity - positive/ATL	Specificity - negative/no-ATL	Accuracy	Comparison with PCR	
	(95% CI)	(95% CI)	(95% CI)	Kappa (95% CI)	p-value*
Smear	50.63% - 40/79	100% - 0/20	60.61%	0.29	< 0.001
	(39.84-61.37)	(83.89-100)	(50.76-69.66)	(0.15-0.43)	
Culture	46.84 - 37/79	100% - 0/20	57.58%	0.26	< 0.001
	(36.34-57.73)	(83.89-100)	(47.74-66.85)	(0.13-0.39)	
Indirect immunofluorescence	69.62% - 55/79	80% - 16/20	71.72%	0.36	1
	(58.77-78.66)	(58.40-91.93)	(62.16-79.65)	(0.19-0.54)	
Amastigotes in histopathology	27.85% - 22/79	100% - 0/20	42.42%	0.13	< 0.001
	(19.17-38.58)	(83.89-100)	(33.15-52.26)	(0.03-0.23)	
Amastigotes, plasma cells, and/or granuloma in histopathology	88.61% - 70/79	50% - 10/20	80.81%	0.39	< 0.001
	(79.75-93.89)	(29.93-70.07)	(71.96-87.36)	(0.19-0.59)	

**ATL:** American tegumentary leishmaniasis, **PCR:** Polymerase chain reaction, **CI:** Confidence interval. *McNemar's test.

**TABLE 3:** Diagnostic accuracy of the Montenegro skin test in the entire population, PCR-negative patients, and those subjected to PCR along with a strongly suggestive histopathology.

	Sensitivity - positive/ATL	Specificity - negative/no-ATL	Accuracy
	(95% CI)	(95% CI)	(95% CI)
PCR in the total population	74.68% - 59/79	100% - 0/20	79.80%
	(64.11-82.97)	(83.89-100)	(70.85-86.52)
Montenegro skin test in total population	82.28% - 65/79	60% - 12/20	77.78%
	(72.42-89.14)	(38.66-78.12)	(68.64-84.84)
Montenegro skin test in PCR (+) patients	79.66% - 47/59		74.68%
	(67.73-87.96)		(64.11-82.97)
Montenegro skin test in PCR (-) patients	90.00% - 18/20		75.0%
	(69.90-97.21)		(59.81-85.81)
Montenegro skin test plus highly suggestive	85.00% - 17/20	80% - 16/20	82.50%
histopathology in PCR (-) patients	(63.96-94.76)	(58.40-91.93)	(68.05-91.25)

**ATL:** American tegumentary leishmaniasis, **PCR:** Polymerase chain reaction, **CI:** confidence interval.

In all subjects, PCR showed a sensitivity and specificity of 74.68% (95% CI = 64.11-82.97) and 100% (95% CI = 83.89-100), respectively, and a diagnostic accuracy of 79.8% (95% CI = 70.85-86.52). On the other hand, the sensitivity, specificity, and accuracy of MST were 82.28% (95% CI = 72.42-89.14), 60% (95% CI = 38.66-78.12), and 77.78% (95% CI = 68.64-84.84), respectively (Table 2).

MST showed 90.0% sensitivity (95% CI = 69.90-97.21) in PCR-negative patients, and this value was 10% higher than the sensitivity value reported in PCR-positive population (79.66%; 95% CI = 67.73-87.96). The evaluation of the samples positive for MST along with highly suggestive histopathological results (positivity for both MST and histopathologic criteria) revealed a better accuracy of 82.5% (95% CI = 68.05-91.25) and an improved specificity of 80% (95% CI = 58.40-91.93). No significant difference was observed in the comparison between only MST and MST + suggestive histopathological exam in PCR-negative patients (p = 0.125).

As part of the presently defined inclusion criteria, all patients with ATL were daily treated with meglumine antimoniate at 20 mg Sb5+/kg, as recommended by the Brazilian Ministry of Health for cutaneous and mucocutaneous forms of ATL^3^. Two patients with mucocutaneous ATL (both presented negative PCR results) and 3 patients with cutaneous ATL (1 presented negative PCR results) were subjected to a similar second course of treatment to ensure complete healing^3^. No influence of PCR result on treatment outcome was detected (p = 0.113). Cure was defined as complete lesion healing after 3 months from the end of the specific treatment^3^.

## DISCUSSION

Considerable investment has been made in molecular biology studies for the diagnosis of ATL, as justified by the enhanced sensitivity and high specificity of these techniques. However, even the most precise techniques do not exhibit complete sensitivity in the clinical setting^8^. Thus, clinicians are required to use other classical immunological tests for the diagnosis of ATL. In the absence of a gold standard diagnostic test, it is important to study the association between the existing tests before the prescription of toxic drugs to patients^2^. Immunological tests such as MST are among the most classic and oldest complementary exams employed for the diagnosis of ATL. Its low specificity and the popularization of molecular biology techniques have reduced the utility of MST. In recent years, Brazil has scarce supplies of the antigen used for MST owing to local regulations. These factors justify the difficulties underlying MST administration and the need for its rational use, given the importance of a test that accurately detects the cellular response to *Leishmania* due to the limitations of PCR.

We hypothesized that one of the main factors that explain the negative PCR result in patients with active ATL is the concomitance of a strong cellular immune response and granuloma formation^13^. This characteristic is frequently reported in chronic cases of ATL as well as in mucocutaneous leishmaniasis but tends to occur in almost all untreated infections caused by *L. braziliensis* ^9^ *.* This is why MST, a cellular response test, was considerably more sensitive in PCR-negative patients (90.0%; 95% CI = 69.90-97.21) than in PCR-positive patients (79.66%; 95% CI = 67.73-87.96). Although serial testing is generally carried out to improve specificity, the use of MST in PCR-negative patients may be beneficial to enhance diagnostic sensitivity, probably owing to biological factors.

This effect was also used to improve the diagnostic accuracy of a rational diagnostic test combination. In general, specificity continues to be limited to 60% (95% CI = 38.66-78.12), which may serve as a limitation when PCR result is negative for suspected leishmaniasis cases. To warrant better specificity, we incorporated a parallel criterion of including highly suggestive histopathological findings (amastigote forms, granuloma formation, and/or plasma cell infiltrates) in MST evaluation. As a consequence, the diagnostic specificity improved (80%; 95% CI = 58.40-91.93) for PCR-negative patients (**Table 2**). However, the sensitivity was slightly reduced to 85% (95% CI = 63.96-94.76).

Non-invasive sampling techniques (swabs and scraps) have been found to be more suitable than tissue biopsies for PCR-based ATL diagnosis^8^ ^,^ ^14^ ^,^ ^15^. We recommend non-invasive PCR for the diagnosis of ATL in regions with the proper structure^16^. Second, in PCR-negative patients with suggestive epidemiological and clinical presentations, the tests that detect immunological cellular responses associated or not associated with histopathology are useful to reduce the rate of false-negative tests.

The main limitation of the present study is that all tests (PCR, histopathology, and MST) were performed at the same time for blinding purposes^17^ ^,^ ^18^. We made the abovementioned recommendations based on a series interpretations from MST result after PCR. However, we believe that the performance of MST only after confirming PCR negativity tends to reveal higher MST values once the disease duration is positively related to the cellular response^2^. A maximum sensitivity of 75% for PCR and 90% for MST may seem insufficient for field diagnosis, but this result is in line with previous studies conducted in this geographical region^7^ ^,^ ^8^. It is also important to remember that cross-sectional accuracy studies result in reduced sensitivity values as compared to other methodologies conducted in well-controlled enviroments^8^ ^,^ ^19^ ^,^ ^20^.

We conclude that one of the most important reasons for PCR negativity in patients with active ATL is the presence of a strong cellular immunological response, especially in cases of chronic and mucocutaneous leishmaniasis. This observation reinforces the use of tests that better detect the cellular response against *Leishmania*, such as MST, in PCR-negative patients because their performance in screening situations is questionable owing to variable accuracy. The analysis of the rational association with other exams such as histopathology is also beneficial. Future studies on other techniques that detect cellular responses in ATL are warranted.
